# Exploring brain lobe-specific insights in an explainable framework for EEG-based schizophrenia detection

**DOI:** 10.1371/journal.pone.0334389

**Published:** 2026-03-20

**Authors:** Md. Milon Hossain, Md. Nurul Ahad Tawhid

**Affiliations:** Institute of Information Technology, University of Dhaka, Dhaka, Bangladesh; University of North Carolina at Chapel Hill, UNITED STATES OF AMERICA

## Abstract

Schizophrenia (ScZ) is a growing global health concern that affects millions of people and puts severe pressure on healthcare systems. Early detection and accurate diagnosis are crucial for adequate management. Electroencephalography (EEG) has evolved into a promising non-invasive tool for detecting ScZ in contemporary research. However, specific biomarkers, especially those related to brain lobes, cannot often be identified by current EEG-based diagnostic methods. Different brain lobes are associated with distinct cognitive functions and patterns of diseases. Also, there is a gap in the incorporation of the explainable artificial intelligence (XAI) technique, as medical diagnosis needs trustworthiness and explainability. This study strives to address these gaps by developing a framework using mel-spectrogram images with Convolutional Neural Networks (CNNs). EEG signals are converted into mel-spectrogram images using Short-Time Fourier Transform (STFT). After that, these images are analyzed using a CNN model to perform classification between ScZ and healthy control (HC). To identify the most critical brain regions, the full brain regions are divided into five different regions, and the same classification process is performed. The performance of the proposed framework is evaluated using two publicly available EEG datasets: repOD and the kaggle basic sensory task dataset, which provides a remarkable accuracy of 99.82% and 98.31% respectively. Among regions, the frontal lobe has the most significant performance with an accuracy of 97.02% and 88.03%, respectively, in these datasets, followed by the temporal lobe. Conversely, the occipital lobe shows the lowest accuracy among lobes, with only 79.30% and 68.33% accuracy on both occasions, showing its lower significance in the diagnosis. To enhance result explainability, three existing XAI technologies—Local Interpretable Model-agnostic Explanations (LIME), SHapley Additive exPlanations (SHAP), and Gradient-weighted Class Activation Mapping (Grad-CAM)—are applied to demonstrate which factors are responsible for the actual outcomes. These findings emphasize the potential of EEG-based brain lobe analysis in enhancing ScZ detection, diagnostic accuracy, explainability, and clinical guidance.

## Introduction

Schizophrenia (ScZ) is a chronic, disabling neuropsychiatric disorder characterized by disturbances in thought, perception, emotions, and behavior. It affects nearly 24 million people, or around 0.33% of the global population, according to the latest World Health Organization (WHO) report [1]. Individuals with ScZ often experience persistent delusions, hallucinations, disordered thinking, cognitive problems, poor social functioning, and reduced quality of life, which cause considerable daily challenges [1]. Although ScZ is a complex disease, it is essential to develop an effective treatment system and enhance patient outcomes. A thorough understanding and management of this complex disease requires a holistic approach that integrates biological, psychological, and social factors [2]. A survey indicated that annual costs for the diagnosis of ScZ patients in the country ranged from US 94 million to US 102 billion, and indirect costs contributed to 50%–85% of the total costs associated with this disease [1]. The increasing frequency imposes a considerable strain on healthcare systems and economies. The economic burden of ScZ was estimated to range from 0.02% to 1.65% of the gross domestic product in most high-income countries. Over the years, it has been increasing rapidly [3,4]. Despite its devastating consequences, ScZ lacks a cure or treatment capable of preventing or stopping its advancement. Nonetheless, early identification can alleviate its consequences and enhance patients’ quality of life. [5].

A few techniques are available for capturing the functional activity of the brain. Conventional diagnostic procedures for ScZ, including clinical evaluations and neuroimaging modalities (MRI, PET scans), are costly, labour-intensive, and not extensively available [6–9]. Consequently, researchers are progressively investigating non-invasive, economical alternatives, such as electroencephalography (EEG) [8–11]. It provides significant benefits due to its superior temporal resolution, cost-effectiveness, user-friendliness, and direct assessment of neural activity [12]. EEG records the brain’s electrical activity using electrodes attached to the scalp [12]. Research has repeatedly demonstrated that persons with ScZ display unique EEG patterns, characterized by modified power spectra, coherence, and connectivity. These correlate with cognitive impairments, sensory processing deficiencies, and other clinical symptoms, especially in several brain lobes [13–15]. The human brain comprises multiple lobes—frontal, temporal, parietal, occipital, and central each essential for cognitive function [13]. Temporal lobe disturbances are frequently linked to memory and language problems in ScZ disease [14], whereas variations in the frontal and parietal lobes may signify cognitive impairment and executive dysfunction [15]. Research has associated EEG abnormalities in particular lobes with neurodegeneration leading to ScZ. There is growing evidence that employing EEG data to identify lobe-specific neural biomarkers could provide an essential aspect in the domain of ScZ diagnosis.

In recent years, numerous studies have concentrated on identifying ScZ by EEG data [16–45]. These studies exploited various machine learning algorithms in conjunction with diverse feature extraction methods for EEG-based ScZ detection. Feature extraction techniques, including time, frequency, and time-frequency analysis [16–18,21], entropy measures [7], connectivity measures [19], complexity measures [20], and event-related analysis [8]. Then these features have been employed alongside established machine learning models such as support vector machines (SVM) [9,22–25], k-nearest neighbours (KNN) [4,26], Boosted trees classifier [27], random forests(RF) [28,29], PSDL [30,31], microstate semantic model [32] and Empirical mode decomposition (EMD) [3]. The ML-based classification techniques described above employ a feature engineering approach to create features from raw EEG data, which demands experts with an in-depth knowledge of the feature domain of interest [46]. Conventional ML techniques, with shallow architectures and limited nonlinear feature adaptation, struggle to capture complex patterns in EEG data. To tackle this difficulty, deep learning (DL) approaches have gained prominence because of their capacity to automatically extract and learn features directly from raw EEG data.

The use of DL in EEG data analysis offers potential for precise diagnosis of symptoms and disease advancement. In their 2021 study, Sun et al. [33] used EEG data from 109 subjects (54 with ScZ, 55 with HC) to demonstrate the effectiveness of fuzzy entropy (FuzzyEn) over the Fast Fourier Transform (FFT). It achieved an average accuracy of 99.22% with FuzzyEn and 96.34% with FFT. The next year, Lillo et al. [18], operating through another dataset of 28 subjects (14 with ScZ, 14 HC), achieved 93% accuracy with a CNN model. Using the same dataset, Oh et al. [36] executed an eleven-layer CNN with 98.07% accuracy in non-subject-based and 81.26% in subject-based testing, and Latreche et al. [34] reached 99.18% accuracy with a ten-layer CNN. Additionally, Yang et al. [35] integrated both deep CNN and fuzzy logic, resulting in a 99.05% accuracy. Compiling on the same dataset of 28 subjects (14 with ScZ, 14 HC), the RDCGRU model was introduced by Sahu et al. [37], which employed several 1-D convolutional layers with lengths greater than one. It attained an accuracy of 88.88% with alpha-EEG rhythms. Additionally, Goker et al. [16] presented a 1D-CNN with multitaper techniques, which increased the accuracy to 98.76% in the same dataset. Guo et al. [45] achieving 99.46% accuracy using a dataset of 28 subjects (14 with ScZ, 14 HC). It further reveals altered brain connectivity and reductions in entropy in the temporal and frontal lobes.

Some studies have transformed EEG into time–frequency images for classification. Using another dataset of 81 samples (49 ScZ and 32 HC), Khare et al. [38] introduced the Continuous Wavelet Transform (CWT), Short-Time Fourier Transform (STFT), and Smoothed Pseudo-Wigner-Ville Distribution (SPWVD) techniques to produce scalograms, spectrograms, and SPWVD-based time-frequency representation (TFR) plots, respectively. The findings demonstrate an accuracy of 93.36% using the SPWVD-based TFR and CNN models. Using the same dataset, Sahu et al. [43] designed a separable convolution attention network (SCZSCAN), which achieved 95% accuracy. Similarly, Aslan et al. [39] utilized STFT-based 2D time-frequency (T-F) images in a DL-based VGG-16 CNN model, which achieved accuracies of 95% and 97.4%, respectively, using two distinct EEG datasets, one of which included 84 subjects (39 HC and 45 ScZ) and the other of which included 28 subjects (14 HC and 14 ScZ). In another study, the same author [40] introduced a method that utilizes the CWT to generate 2D T-F scalogram images. The same VGG16 CNN model was used, achieving accuracies of 98% and 99.5% on previously used datasets. Moreover, techniques such as Activation Mapping, Saliency Map, and Grad-CAM are employed to visualize learning results. Beyond this, using a dataset of 84 individuals (45 ScZ, 39 HC), Naira et al. [41] employed the Pearson correlation coefficient (PCC) with CNNs, achieving an accuracy of 90%. Li et al. [42] applied a lightweight Vision Transformer model (LeViT) with CNN, which obtained a subject-independent performance of 98.99%.

Although some have reached various levels of significance in some cases, their ability for robustness and generalization is limited. None of the previous approaches considered brain lobe-specific diagnosis of ScZ, as different brain lobes are responsible for various functionalities in the human brain. According to recent studies, recognizing lobe-specific EEG biomarkers can enhance the improvement of the diagnosis process [13–15].

In medical decision-making systems, explainable artificial intelligence (XAI) has become an essential element for diagnosing neuropsychiatric disorders, as medical diagnoses require trustworthiness and explainability [47]. Deep learning models offer robust tools for automated classification, but their “black-box” nature limits their interpretability and clinical trust. XAI can bring transparency by highlighting relevant abnormalities that impact model predictions, thereby helping to build reliable diagnostic tools and increasing clinician confidence [48]. Our goal in incorporating XAI into deep learning models is to identify the reasons behind classification decisions. Most importantly, focusing on lobe-specific EEG modifications, we aim to enhance diagnostic precision and aid in the development of non-invasive, accessible, and reliable instruments for the early detection of ScZ.

In this study, to address the existing research gap, we have employed a framework for detecting ScZ and evaluated which brain region is most influential in diagnosis. First, by performing some preprocessing steps using STFT, the EEG signals were converted into mel spectrogram. Then, the converted mel-spectrogram images are fed into CNN models with 10-fold cross-validation. Following the same methodology, we classified the mel spectrograms from each brain lobe and the entire EEG channel set independently to assess how well each approach worked for identifying ScZ. The results obtained from the proposed methods are compared with those from other existing literature that used the same dataset. Notably, three XAI techniques are implemented to introduce explainability to the proposed framework and its outcomes. This research contributes to these noteworthy contributions as follows:

- **Novel biomaker development:** This research introduces a novel framework that integrates STFT-based mel-spectrogram images with a CNN model that distinguishes between ScZ and HC using EEG data.
- **Analysis of brain regions:** It explores which brain regions are essential for extracting representative information for accurate ScZ detection.
- **Improvement of performance:** The study seeks to improve the performance of ScZ detection relative to existing methodologies.
- **XAI integration:** The study utilizes SHAP, LIME, Grad-CAM to explain the model’s decision-making process, highlighting why different brain regions perform differently in ScZ detection

The following sections of this study are organized as follows: the “Methodology” section outlines the data studied and the proposed method, experimental setup, pre-processing steps, etc. The “Results and Discussion” section combines both components; it presents the result outcomes and thoroughly analyzes the experimental findings. Then, the explainability of the proposed method is discussed by applying XAI techniques. Finally, the “Conclusions” section summarizes the paper and addresses the scope for future study.

## Methodology

This study presents a framework for identifying significant brain lobes associated with the detection of ScZ, utilizing EEG brain signal data. The article introduces an approach that integrates STFT-based mel-spectrogram and CNN to identify critical brain lobes as biomarkers. The system initiates with the preprocessing of EEG signals, applying a butterworth band-pass filter to reduce noise. The EEG data are subsequently divided into smaller time segments. Afterwards, the EEG channels are divided into five brain lobes according to biological principles. Mel-spectrogram images are produced for each brain lobe and a full range of EEG channels with STFT, which offers a time-frequency representation of cerebral activity. The mel-spectrograms are then input into a DL-based CNN to classify ScZ vs HC. The classification is conducted independently on the mel-spectrogram images from each brain lobe and the full channel group. All experiments were implemented using the Python programming language. The deep learning models were developed using the TensorFlow/Keras framework. Data preprocessing and signal processing were performed using NumPy, SciPy, and MNE-Python libraries. The mel-spectrogram generation utilised the Librosa library. For explainability analysis, we employed the SHAP, LIME, and tf-keras-vis (for Grad-CAM) packages. Data visualisation was conducted using Matplotlib and Seaborn libraries. Explainability techniques determine which input regions affect predictions for CNN-based image classification. To highlight key areas, LIME modifies image regions and tracks changes in predictions [49,50]. Using game theory, SHAP determines the contribution of each pixel and assigns relevance scores that indicate which regions influence the classification choice [49]. Grad-CAM produces a heatmap that highlights the spatial areas that CNN concentrated on using gradients from the predicted class [51]. Details of these steps are discussed below:

### Proposed Framework

**Fig 1:**
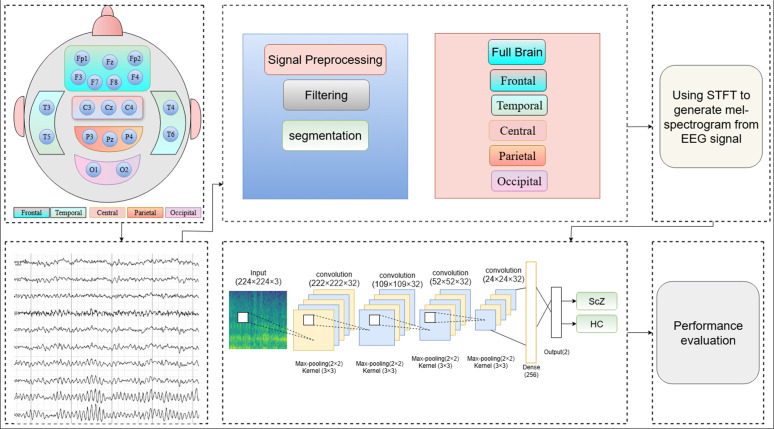
Overview of the proposed framework for detecting ScZ using EEG data.

The integration of STFT based mel-spectrogram images with CNN is highly effective for ScZ detection, as STFT encompasses both the time and frequency domains of the brain’s electrical activity, providing valuable insights into cognitive decline. CNNs are recognized for their ability to identify patterns in 2D data such as mel-spectrograms, excel at detecting complex and sophisticated features related to ScZ. This strategy aims to more effectively detect ScZ-related changes in brain wave patterns. A summary of the proposed framework is shown in Fig 1, with comprehensive details available in the following subsections.

### Dataset description

In our study, two publicly available EEG datasets are used for testing. The first group of data is publicly available called as the repOD dataset and was gathered from the Institute of Psychiatry and Neurology in Warsaw [19]. This dataset includes 19-channel resting state EEGs collected from 28 volunteers of similar ages and genders, half of whom are healthy controls (HC = 14) and the other Half (ScZ = 14) showed signs of ScZ. The Second dataset, which is comparatively underexplored, was obtained as part of a project financed by the National Institute of Mental Health (NIMH; R01MH058262). This dataset is available to the public on the Kaggle platform [52]. The dataset consists of 64-channel scalp EEG data gathered from 81 Subjects, composed of 49 with ScZ and 32 HCs. A summary of these two datasets is provided in the Table 1.

**Table 1:** Details of publicly available EEG Schizophrenia dataset.

Key details	RepOD dataset [53].	Kaggle basic sensory task dataset [52].
Total subjects	28	81
Healthy control (HC) and ScZ Subjects	(14, 14)	(32, 49)
Male and Female ScZ Subjects	(7, 7)	(41, 8)
Male and Female HC Subjects	(7, 7)	(26, 6)
Mean age (HC) in years	27.75 ± 3.15 years	40.02 ± 13.48 years
Mean age (ScZ) in years	28.1 ± 3.7 years	38.37 ± 13.91 years
Mean age (Male HC) in years	26.8 ± 2.9 years	38.15 ± 12.97 years
Mean age (Male ScZ) in years	27.9 ± 3.3 years	40.21 ± 12.93 years
Mean age (Female HC) in years	28.7 ± 3.4 years	39.33 ± 18.91 years
Mean age (Female ScZ) in years	28.3 ± 4.1 years	39 ± 16.98 years
No. of EEG Channels	19	64
Recording Duration	15 min (Approx.)	1 s data for each conditions from 1 to 3
Sampling Frequency	250	1024

In the repOD dataset, all subjects were placed in an eyes-closed resting state condition, and their EEG data were captured for 15 minutes. The conventional 10–20 EEG setup with 19 EEG channels, including Fp1, Fp2, F7, F3, Fz, F4, F8, T3, C3, Cz, C4, T4, T5, P3, Pz, P4, T6, O1, O2, was used to collect the data at a sampling frequency of 250 Hz. FCz was the location of the reference electrode. The basic sensory dataset consists of 64 channels, with each file containing 74 columns. The first four columns describe the data, including subject, trial, sample, and condition. The last six columns represent reference/EOG electrodes, such as VEOa, VEOb, HEOL, HEOR, Nose, and TP10. The 64 EEG channels are as follows: ‘Fp1’, ‘Fpz’, ‘Fp2’, ‘AF7’, ‘AF3’, ‘AFz’, ‘AF4’, ‘AF8’, ‘F7’, ‘F5’, ‘F3’, ‘F1’, ‘Fz’, ‘F2’, ‘F4’, ‘F6’, ‘F8’, ‘FC5’, ‘FC3’, ‘FC1’, ‘FCz’, ‘FC2’, ‘FC4’, ‘FC6’, ‘C5’, ‘C3’, ‘C1’, ‘Cz’, ‘C2’, ‘C4’, ‘C6’, ‘FT7’, ‘T7’, ‘TP7’, ‘FT8’, ‘T8’, ‘TP8’, ‘P7’, ‘P8’, ‘P9’, ‘P10’, ‘CP5’, ‘CP3’, ‘CP1’, ‘CPz’, ‘CP2’, ‘CP4’, ‘CP6’, ‘P5’, ‘P3’, ‘P1’, ‘Pz’, ‘P2’, ‘P4’, ‘P6’, ‘O1’, ‘Oz’, ‘O2’, ‘Iz’, ‘PO7’, ‘PO3’, ‘POz’, ‘PO4’, ‘PO8’. The dataset includes EEG recordings from three experimental conditions: Condition(1), button press to generate a tone, records neural signals during motor execution combined with self-generated auditory feedback, reflecting sensorimotor integration. Condition (2), passive listening to tone, captures pure auditory processing of external stimuli without motor involvement, isolating sensory perception. Condition (3), button press without tone, records motor-related brain activity alone, serving as a control to separate motor signals from auditory processing.

Participants’ anonymity of both datasets are ensured by withholding any personally identifiable information from publication.

### Experimental Setup

In the experiment, we used two datasets, both containing two categories of subjects, and carried out classification tasks with ScZ and HC. The basic sensory dataset consisted of 49 patients with ScZ and 32 with HCs. We have used a segmentation length of 3-s, consistent with the approach used in prior research [6,54–56]. Then, we have generated the mel-spectrogram images from those signal segments using STFT and mel filter bins. After these segmentation and image generation steps, the resulting dataset contains 9610 and 7713 images, respectively. The repOD dataset provided 4339 images of HC and 5271 images of ScZ, while the basic sensory dataset ended up with 3102 HC and 4611 ScZ images. The dimensions of those images are 224×224 pixels and are then used as input for the CNN model. The same methodology is applied to several brain regions to produce mel-spectrogram images using the channel data from those areas, ensuring consistency and facilitating analyses of the brain lobes. The studies are performed on a PC with a configuration setup that included 128 GB of RAM and a 16 GB of graphics memory, providing sufficient resources for effective processing and analysis. We have performed 100 epochs and a training batch size of 32 to train the CNN model. Additionally, mel-scale frequency bands 112, actual window length-256 and FFT window size-512 are used for the experiment.

### Pre-processing EEG Signals

The EEG signal is preprocessed in several ways to improve its quality and get it ready for analysis. The same preprocessing pipeline is applied across both of the datasets.

### Noise removing

In repOD, a butterworth filter of order 2 was implemented to filter each EEG channel’s signals in the physiological frequency bands listed below: Delta is 2–4 Hz, theta is 4.5–7.5 Hz, alpha is 8–12.5 Hz, beta is 13–30 Hz, and gamma is 30–45 Hz. In kaggle dataset, EEG data underwent preprocessing with a 0.1 Hz high-pass filter, outlier interpolation for noise reduction, re-referencing to the average of the ear lobes, Single-trial epochs were separated, and baseline correction was implemented. Outliers were eliminated, components were extracted by independent component analysis, and noise was mitigated using canonical correlation analysis. Artefacts (e.g., eye blinks, muscle movements) are removed automatically, and after removing these, by analog to decimal conversion, data is already given in the dataset against the respective channel [19,52]. These measures guaranteed data that is clean and prepared for analysis. Re-referencing is handled with a 10–20 international electrode position system in EEG. Then, for these two datasets, a butterworth band-pass filter ranging from 0.5 to 45 Hz was applied to enhance signal quality according to the prior research [9,31].

### Resampling

The signals were resampled to 256 Hz to normalize the data, ensuring consistency in the sampling rate across all channels and enabling accurate comparison and analysis between these two datasets. It is the standard practice in prior research, as sampling is usually done in $2^{n}$ times [24,38].

### Segmentation

In our investigation, the signals were eventually segmented to increase the dataset size by dividing the signal data into equal time intervals and each segment is assigned the same label as the original sample [6,39]. EEG data were segmented into 3-second segments, as is a standard practice in earlier studies [6,38,54–56]. These small portions help capture essential patterns in brain activity without introducing excessive amounts of irrelevant information. This enables the model to extract important features from the signal. Additionally, shorter segments speed up processing and reduce the computational load by decreasing the amount of data that computer must process simultaneously, while preserving essential information.

### Brain Lobe Schedule

**Fig 2:**
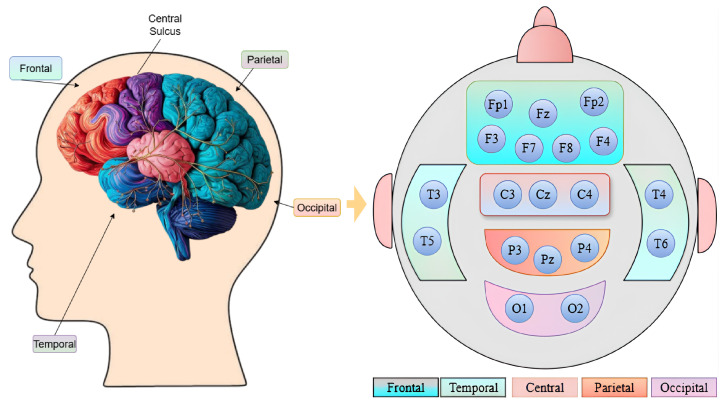
An illustration of the brain lobe structure showing the arrangement of EEG channels for repOD Dataset.

To explore the significance of each brain region, we organize the EEG channels into the various brain lobes. The electrodes are positioned according to the 10–20 system, a standardized approach that assures accurate and uniform placement on the scalp based on anatomical landmarks, as shown in Fig 2. The frontal region, linked to higher thinking and decision-making, is represented by Fp1, Fp2, F3, F4, F7, F8, and Fz. The temporal region, associated with memory and auditory processing, included T3, T4, T5, and T6. The occipital region, responsible for visual processing, used O1 and O2. The central region, involved in motor control and sensory integration, is represented by C3, C4, and Cz. The parietal region, related to spatial orientation, included P3, P4, and Pz.

**Fig 3:**
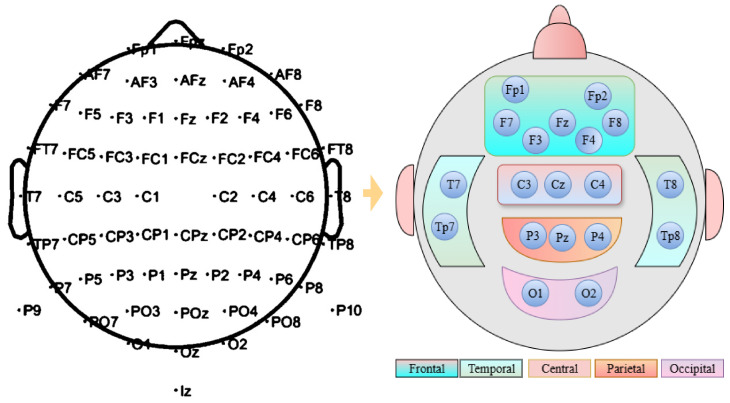
An illustration of the brain lobe structure showing the arrangement of EEG channels for kaggle basic sensory dataset from 64 channels to 19 channels.

The repOD dataset consists of 19-channel electrodes. For the Kaggle dataset, we selected an identical position for 19 channels out of the 64 channels, according to the 10–20 international electrode position system. The 10–20 system, so named because electrodes are placed at intervals of 10% or 20% of the distance between important brain landmarks (nasion, inion, and preauricular points), is a globally defined technique for applying EEG electrodes to the scalp [6,24]. Electrodes are tagged with numbers that indicate hemisphere position (odd = left, even = right, z = midline), such as F3, C4, or Pz, and letters that indicate brain areas (F = frontal, C = central, T = temporal, P = parietal, O = occipital). This standardized placement ensures consistent electrode positioning across different individuals and studies, enabling reliable comparison of EEG data and accurate identification of brain activity in specific regions. By maintaining this system, we chose 19 channels from 64 channels. Then, we divided it into five regions. For the frontal region, the following channels are selected: Fp1, Fp2, F7, F3, Fz, F4, F8. Then, the central region is represented by channels ‘C3’, ‘Cz’, and ‘C4’. The temporal and parietal regions are represented by: T7, T8, Tp7, Tp8, and P3, Pz, P4, respectively. Lastly, the occipital region is followed by the channels O1 and O2. Then, all 19 channels are examined as a single set to cover the full region. Channel selection from 64 channels to 19 channels is shown in Fig 3. By organizing the channels based on brain regions, the focus is more precise on how each area contributes to the signals.

### Converting EEG Signals to mel-spectrogram Images

A Mel-spectrogram is a spectrogram in which the frequencies are transformed to the mel scale [51]. The Mel-spectrum comprises an STFT for each spectrum frame (energy/amplitude spectrum), changing from a linear frequency scale to a logarithmic Mel scale, afterwards processed through a filter bank to obtain the eigenvector; these eigenvalues can be approximately represented as the distribution of signal energy across the Mel-scale frequencies. To achieve this, it is initially necessary to calculate the STFT of the signal to convert it into mel-spectrogram images [51,57]. The STFT is an effective signal processing method used for analyzing the frequency elements in non-stationary signals over time [6]. Following that, the Fourier transform is carried out on each segmented window, producing its specific frequency spectrum. The procedure involves converting the time-varying EEG signal into a two-dimensional (2D) matrix, where time is represented on the horizontal axis and frequency is represented on the vertical axis. The horizontal axis represents time, with the EEG signal divided into segments or windows. The vertical axis denotes the frequency range, generally starting with lower frequencies at the bottom and progressing to higher frequencies at the top [6]. A Hamming window is used for each segment to maintain continuity and minimize spectral leakage. The STFT of a signal at a specific time t and frequency f, denoted as STFT(t, f), is calculated using the following equation:


$$X(t,f)=\int_{-\infty }^{\infty }x(\tau )\;w(\tau -t)\;e^{-j2\pi f\tau }\;d\tau$$ (1)

The next phase involves applying a mel filter bank to the STFT output. The mel scale is a logarithmic scale that reflects frequency perceptions, with increased sensitivity to lower frequencies. The mel scale is defined by the following equation:


$$mel(f)=2595\log_{10}\left(1+\frac{f}{700}\right)$$ (2)

A mel filter bank has overlapping triangular filters that align with various mel frequencies. The filters are placed closely for lower frequencies and more widely for higher frequencies, matching the mel scale [51]. The logarithmic transformation compresses the amplitude range and highlights the more important features of the signal. The outcome is a mel-spectrogram image, a time-frequency representation in which the frequency axis has been converted from linear to mel frequency [57]. The mel-spectrogram illustrates the spectrum of a signal’s energy over time and frequency [51,57].

### Classification of Schizophrenia: Training the CNN Model with Spectrogram Images

**Fig 4:**
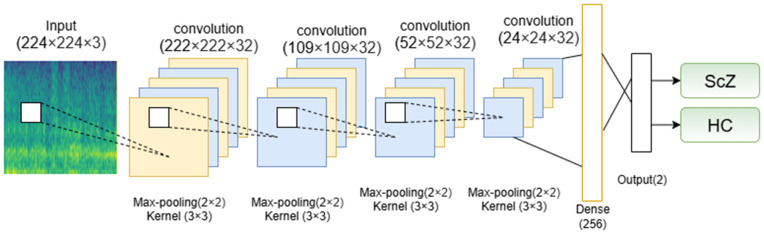
Architecture of the CNN model used in this study.

For the classification using mel-spectrogram images, we used a established CNN model from prior research [6]. CNNs are well-known in deep learning for their exceptional efficiency in the execution of classification tasks [16,34,45,54]. They excel in this field by independently finding and extracting relevant features from the input images, thus facilitating accurate classification into several categories [54,58]. Here is the used CNN model architecture in Fig 4.

CNNs accumulate features gradually, beginning with basic patterns and advancing to complex ones. This layered learning process enhances their ability to understand and classify complex visual information with greater precision. When utilized on spectrogram images, adding Mel-mins, which visually represent EEG signals, CNNs can accurately identify and classify them into appropriate groups. Due to their ability to identify complex patterns, CNNs are highly effective and advantageous for numerous image classification tasks [54,59].

CNNs achieve their efficiency through the specific configuration of their layered architecture. Each convolutional layer contributes individually to feature extraction, enabling the network to learn visual representations hierarchically [6]. Primarily, the layers identify low-level features, including edges, corners, and basic textures. While these features may seem insignificant individually, they constitute the essential components for complex representations. As the data advances through successive layers, the network progressively synthesizes these basic elements into more abstract and semantically significant features [55,58]. Combinations of edges and textures may create simple geometric shapes that can then become elements in larger objects. As a result, deeper layers in the network can identify high-level patterns and abstract concepts essential to differentiating objects in an image, which enables CNNs to surpass the perception of visual data as merely a structured collection of pixels. The network promotes a systematic and hierarchical approach to interpreting imagery, as it proficiently encodes both local details and global representations of visual content [54,58].

The convolution process is fundamental in CNNs, as it is essential for extracting significant features from images, thereby facilitating tasks such as image classification and object detection. In a CNN, the input data are processed using 2-D kernels, where convolution is performed by sliding the kernels over the spatial dimensions and computing the sum of dot products between the kernel and the input [6]. The resulting feature maps are then passed through an activation function to introduce non-linearity. The activation value at spatial position (x,y) in the $j^{\text{th}}$ feature map of the $i^{\text{th}}$ layer, denoted as $v_{x,y}^{i,j}$, is computed as follows:


$$v_{i,j}^{x,y}=\phi \left(b_{i,j}+\sum_{\tau =1}^{d_{i-1}}\sum_{\rho =-\gamma }^{\gamma }\sum_{\sigma =-\delta }^{\delta }w_{i,j,\tau }^{\sigma ,\rho }\times v_{i-1,\tau }^{x+\sigma ,\;y+\rho }\right)$$ (3)

Here, *ϕ* denotes the activation function, and $b_{i,j}$ is the bias term for the $j^{\text{th}}$ feature map in the $i^{\text{th}}$ layer. The term $d_{i-1}$ represents the number of feature maps in the $(i-1{)}^{\text{th}}$ layer, which also corresponds to the depth of the kernel $w_{i,j}$. The kernel dimensions are defined by (2γ+1)×(2δ+1, where 2γ+1 is its width and 2δ+1 is its height. Finally, $w_{i,j,\tau }^{\sigma ,\rho }$ represents the weight parameter of the kernel for the $j^{\text{th}}$ feature map in the $i^{\text{th}}$ layer, while $v_{i-1,\tau }^{x+\sigma ,y+\rho }$ refers to the activation value from the previous layer.

This equation executes a convolution operation by computing the dot product of the filter weights and a localized portion of the input feature map. Integrating the bias, the activation function is employed to produce the final output. The used CNN model aims to effectively extract and learn significant features from input images using a sequence of convolutional and pooling layers. Each convolutional layer uses 32 filters of dimensions 3×3, facilitating the network’s ability to recognize complex spatial patterns. In contrast, max pooling layers with a 2×2 filter are implemented to systematically reduce spatial dimensions and computational complexity without sacrificing critical information. Dropout layers with a rate of 25% are intentionally implemented after the second and fourth convolution-pooling pairs to mitigate overfitting and improve model stability. The combination of convolutional, pooling, and dropout layers assures effective feature extraction while preserving the model’s generalization capability [6]. Then, the network advances to a fully connected (dense) layer including 256 units to combine the acquired features into enhanced representations. A dropout rate of 50% is implemented prior to the final output layer to mitigate overfitting. The classification uses a softmax activation function, generating probabilities for the two target groups: ScZ vs Hc. The model employs categorical cross-entropy loss for training and utilizes the Adam optimizer, resulting in consistent and efficient learning outcomes. This architecture provides a balanced approach to feature learning, regularization, and classification, making it highly suitable for addressing the defined issue.

The detailed breakdown of the layer configurations is shown in Table 2.

**Table 2:** The configuration of the CNN model used in this study.

Layer type	Filters/units	Kernel size	Strides	Activation	Padding	Dropout rate
Input	–	–	–	–	–	–
Conv2D	32	3×3	1×1	ReLU	Same	–
MaxPooling2D	–	2×2	2×2	–	–	–
Conv2D	32	3×3	1×1	ReLU	Same	–
MaxPooling2D	–	2×2	2×2	–	–	–
Dropout	–	–	–	–	–	0.25
Conv2D	32	3×3	1×1	ReLU	Same	–
MaxPooling2D	–	2×2	2×2	–	–	–
Conv2D	32	3×3	1×1	ReLU	Same	–
MaxPooling2D	–	2×2	2×2	–	–	–
Dropout	–	–	–	–	–	0.25
Flatten	–	–	–	–	–	–
Dense	256	–	–	ReLU	–	–
Dropout	–	–	–	–	–	0.50
Dense(Output)	2	–	–	Softmax	–	–

### Performance Evaluation Methods and Metrics

The main objective of our proposed framework is to accurately assess whether a generated image reflects a HC or one with ScZ, and to figure out which brain region most significantly influences the classification process. For doing so, this research empirically assesses the validation performance through a CNN model. This method facilitates obtaining a trustworthy performance assessment while reducing the possibility of overfitting or underfitting. In standard cross-validation(CV), the training and testing sets vary in each iteration, ensuring that every data point is evaluated. To ensure thorough evaluation and enhanced accuracy, we have opted for a 10-fold CV for our proposed framework. Then, Normalization is introduced to ensure that all input attributes, specifically the pixel values of images, fall within an equivalent range. In this process, each pixel value is divided by 255, resulting in a new range between 0 and 1. It helps in reducing the chances of issues related to differences in scale among input features, ensuring that all data points are treated equally during the model’s training.

To evaluate the model, a series of performance tests is conducted, and the results are analyzed. To thoroughly investigate the efficiency of the proposed solution and methodology, we quantified six established metrics: specificity (Spec), sensitivity (Sen), precision (Prec), accuracy (Acc), F1 score (F1), and false positive rate (FPR). Furthermore, a receiver operating characteristic (ROC) curve serves as a graphical equipment to evaluate the diagnostic performance of the binary classifier system. All metrics are calculated using already established formulas [6,24]. A ROC curve is a visual representation that is operated to assess the diagnostic accuracy of a binary classification system. It graphs the true positive rate (sensitivity) vs the false positive rate over several threshold settings. The ROC curve is a valuable tool in medical research and machine learning to evaluate a model’s ability to distinguish between two categories, such as the presence or absence of a disease. This enables it to be a crucial tool for assessing predicted precision in various fields, particularly in diagnostic tests and classification challenges [6,58].

## Results and discussion

This study has proposed a comprehensive framework to identify the essential brain lobes implicated in EEG signal data for the identification of ScZ. The main aim was to assess and compare the effectiveness of this framework in differentiating between individuals with ScZ and HC. Two datasets were analyzed in this study: the RepOD dataset and the Kaggle basic sensory task dataset. Detailed descriptions of these datasets and their preprocessing are provided in the “Experimental Setup” subsection of the Methodology section. The framework underwent evaluation through a 10-fold CV process to ascertain the robustness and generalizability of the findings. Then, we applied LIME, SHAP, and Grad-CAM for the explainability of the ScZ diagnosis. Then, the same method is followed across various brain lobes to see which regions most significantly contributed to differentiating ScZ patients from HCs in classification tests.

### Brain Region-Specific Findings

Tables 3 and 4 show the 10-fold CV average result of the experiments for various brain lobes for the two considered datasets in this study.

**Table 3:** Brain Lobes Classification Results – RepOD dataset.

Metrics	Full brain	Central	Frontal	Occipital	Parietal	Temporal
Sensitivity	99.91±0.13	91.39±1.85	97.65±0.70	86.30±4.76	91.24±1.21	96.71±1.00
Specificity	99.72±0.20	90.91±1.46	96.27±0.65	70.36±23.64	90.58±1.34	95.99±0.94
Precision	99.77±0.16	92.45±1.04	96.95±0.56	79.88±7.90	92.16±1.16	96.67±0.89
F1	99.84±0.12	91.90±0.90	97.29±0.33	82.47±3.49	91.69±0.75	96.69±0.62
Accuracy	99.82±0.13	91.19±0.80	97.02±0.34	79.30±7.61	90.94±0.74	96.37±0.66
FPR	0.28±0.20	9.09±1.46	3.73±0.65	29.64±23.64	9.42±1.34	4.01±0.94

Table notes: Performance metrics for brain lobe classification across different brain regions. Bold values indicate the highest accuracy scores. Results show the mean ± standard deviation in 10-fold cross-validation.

**Table 4:** Brain Lobes classification Results – basic sensory kaggle dataset.

Metrics	Full brain	Central	Frontal	Occipital	Parietal	Temporal
Sensitivity	98.89±0.31	82.08±2.04	91.24±2.40	78.49±4.58	79.83±2.58	81.71±4.03
Specificity	97.43±1.13	54.57±4.90	83.19±3.53	53.02±5.46	51.65±5.22	61.05±5.26
Precision	98.31±0.66	72.93±2.12	89.07±1.62	71.37±2.09	71.11±2.54	75.82±1.64
F1	98.60±0.33	77.20±1.39	90.11±1.17	74.68±2.40	75.15±1.34	78.59±1.87
Accuracy	98.31±0.41	71.04±1.77	88.03±1.45	68.33±1.66	68.46±1.92	73.47±1.57
FPR	2.57±1.13	45.43±4.90	16.81±3.53	46.98±5.46	48.35±5.22	38.95±5.26

Table notes: Performance metrics for brain lobe classification across different brain regions. Bold values indicate the highest accuracy scores. Results show the mean ± standard deviation in 10-fold cross-validation.

Table 3 highlights the performance of our proposed framework for the ScZ vs. HC classification on the repOD dataset. It reports that, when considering the full brain region, we achieved a notable accuracy of 99.82%, with a minimal standard deviation of ±0.13. For the Kaggle dataset in the Table 4 shows, the framework performed slightly lower but still achieved a strong accuracy of 98.31%, with a standard deviation of ±0.41, which is one of a least explored datasets for ScZ diagnosis. We also examined the framework’s performance across individual brain lobes to determine how specific regions contributed to the overall classification accuracy. The accuracy varied among the five brain lobes, with the Occipital lobe showing the lowest performance at 79.30 ± 7.61 and 68.33 ± 1.66 on both datasets. Then, it was followed by the Parietal lobe at 90.94 ± 0.74 and 68.46 ± 1.92 on both occasions. The Central lobe achieved an accuracy of 91.19 ± 0.80 and 71.04 ± 1.77, which is slightly better than the Parietal lobe, while the temporal lobe performed better at 96.37 ± 0.66 and 73.47 ± 1.57 both times. Notably, the frontal lobe yielded the highest accuracy at 97.02 ± 0.34 and 88.03 ± 1.45, as provided on the different occasions.

**Fig 5:**
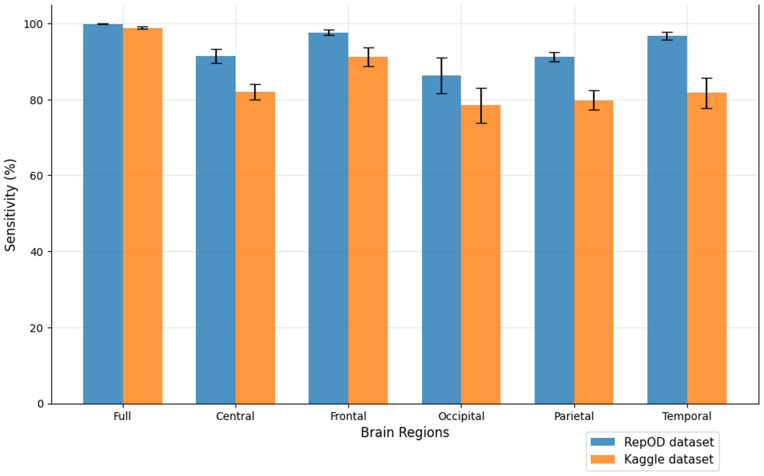
Sensitivity comparison across different brain regions and dataset.

**Fig 6:**
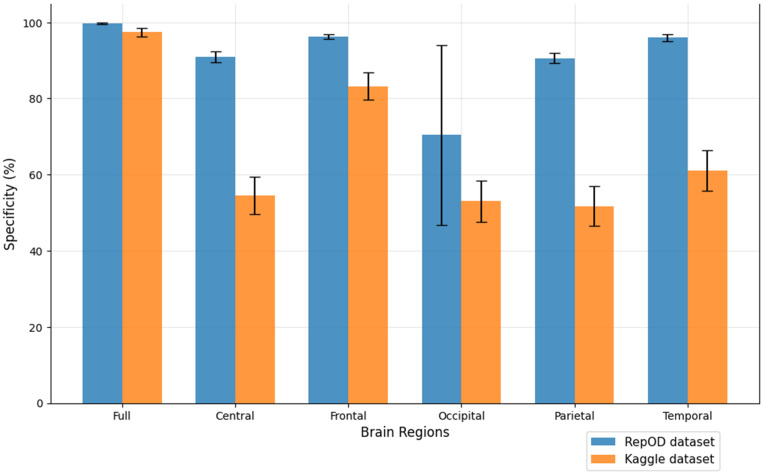
Specificity comparison across different brain regions and dataset.

**Fig 7:**
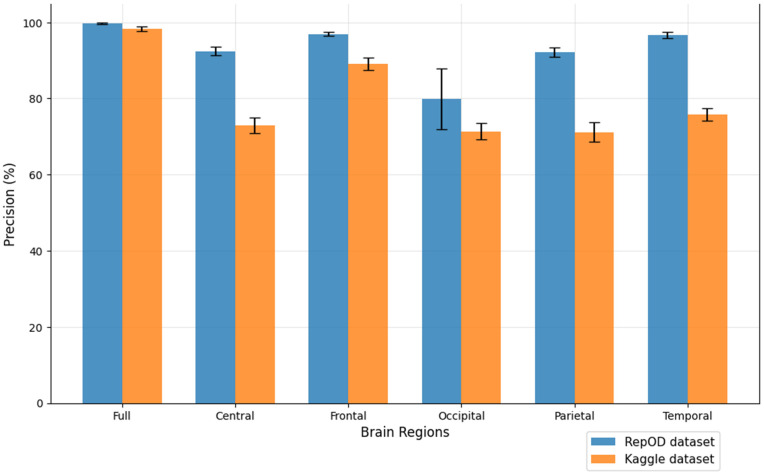
Precision comparison across different brain regions and dataset.

**Fig 8:**
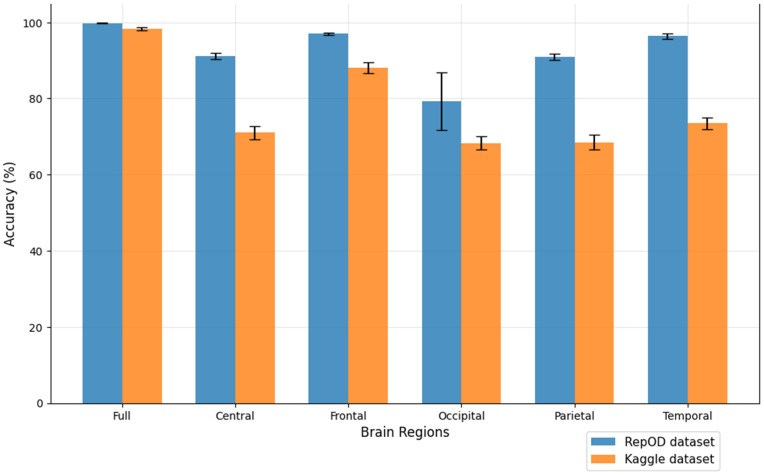
Accuracy comparison across different brain regions and dataset.

To improve our understanding of the performance of several brain lobes across different evaluation metrics, we visually contrasted sensitivity, specificity, precision, and accuracy for each lobe with error bars. Fig 5–8 illustrate these variations, offering an accurate representation of the performance across the two datasets. Since we constructed the performance metrics on two datasets, it is important to visualize the contribution of each brain lobe across the datasets. These visualizations clarify the variety in performance measures throughout the brain lobes.

Figs 5–8 present a comprehensive comparison of classification performance metrics—sensitivity, specificity, precision, and accuracy, respectively—across different brain regions for both the repOD and Kaggle datasets. The full brain region consistently performs exceptionally well, exhibiting its comprehensive diagnostic potential with a sensitivity of 99.91% (repOD) and 98.89% (Kaggle), a specificity of 99.72%, a precision of 99.77%, and an accuracy of 99.82% (repOD) and 98.31% (Kaggle). The frontal region is the most consistent performance among individual lobes in both datasets. It has a significant specificity of 96.27%, accuracy of 97.02% (repOD) and 88.03% (Kaggle), and high sensitivity of 97.65% (repOD) and 91.24% (Kaggle). These results are consistent with previous research on frontal lobe impairments in executive function and cognitive processing and highlight the frontal lobe’s crucial role in ScZ detection. The temporal lobe shows strong performance in the repOD dataset with a sensitivity of 96.71%, a specificity of 95.99%, and an accuracy of 96.37%, ranking second among individual lobes. These results, however, significantly decrease to 81.71% and 73.47% in the Kaggle dataset, suggesting dataset-specific variability that might be caused by variations in recording procedures or participant characteristics.

The central and parietal regions demonstrate moderate and comparable performance, with sensitivity around 91%, specificity around 90–91%, precision around 92%, and accuracy around 91% in the repOD dataset, though performance decreases in the Kaggle dataset to approximately 68–82% across metrics. The occipital region consistently underperforms across all metrics in both datasets. It shows the lowest sensitivity of 86.30% (repOD) and 78.49% (Kaggle), suggesting its difficulty in accurately identifying patients with ScZ. More importantly, it exhibits exceptionally low specificity of 53.02% (Kaggle) and 70.36% (repOD), which leads to large false positive rates and limited ability to differentiate healthy controls. Additionally, the occipital region has the lowest accuracy of 68.33% (Kaggle) and the lowest precision of 79.88% (repOD), indicating that the neural characteristics most pertinent to ScZ disease may not be adequately captured by the occipital lobe.

Then we performed a one-way analysis of variance (ANOVA) followed by paired t-tests and Bonferroni-corrected post-hoc comparisons to assess the regional performance differences of the brain. The F-statistic denotes the test statistic from ANOVA (Analysis of Variance). The t-statistic denotes the test statistic from paired t-tests. The p-value represents the probability of obtaining results at least as extreme as those observed, assuming no real difference exists. Alpha (*α*) represents the significance level, which is the threshold for deciding statistical significance. One-way analysis of variance (ANOVA) revealed highly significant differences in classification performance among brain regions for both datasets (RepOD: F = 38.48, p<0.0.001; Kaggle: F = 236.83, p<0.0.001), providing strong evidence that regional selection significantly impacts ScZ detection accuracy. The frontal lobe significantly outperformed temporal (0.65% advantage, t = 2.28, p = 0.048), central (5.84%, t = 22.17,p<0.0.001), parietal (6.12%, t = 59.59, p<0.0.001), and occipital regions (17.72%, t = 7.00, p<0.0.001), with all comparisons except frontal vs temporal surviving Bonferroni correction (adjusted *α* = 0.005). In Kaggle, frontal superiority was more pronounced, with advantages of 14.61% over temporal (t = 17.31, p<0.0.001), 16.98% over central (t = 34.91, p<0.0.001), and 19.61% over both parietal (t = 53.78, p<0.0.001) and occipital regions (t = 22.41, p<0.0.001), all surviving multiple comparison correction. The frontal lobe’s superior diagnostic value for ScZ classification across both datasets is confirmed by the high t-statistics and tight confidence intervals. Then, in RepOD, occipital accuracy was significantly lower than all other regions, with deficits of 17.72% vs frontal (p<0.0.001), 17.07% vs temporal (t = 6.85, p<0.0.001), 11.89% vs central (t = 4.55, p = 0.001), and 11.60% vs parietal (t = 4.56, p = 0.001), all maintaining significance after Bonferroni correction. In Kaggle, occipital performance showed a 19.61% deficit vs frontal (p<0.0.001) and 5.00% vs temporal (t = 4.18, p = 0.002), with no significant difference from parietal regions (t = 0.00, p = 1.000). The consistently poor occipital performance across datasets, validated through ANOVA, paired t-tests, and Bonferroni-corrected comparisons, indicates that occipital EEG signals provide minimal diagnostic value for ScZ detection. These results are consistent with neurobiological findings that ScZ mostly impacts executive function in frontal regions rather than visual processing in the occipital cortex, supporting the importance of frontal lobe signals while showing little significance of occipital signals [60,61].

**Fig 9:**
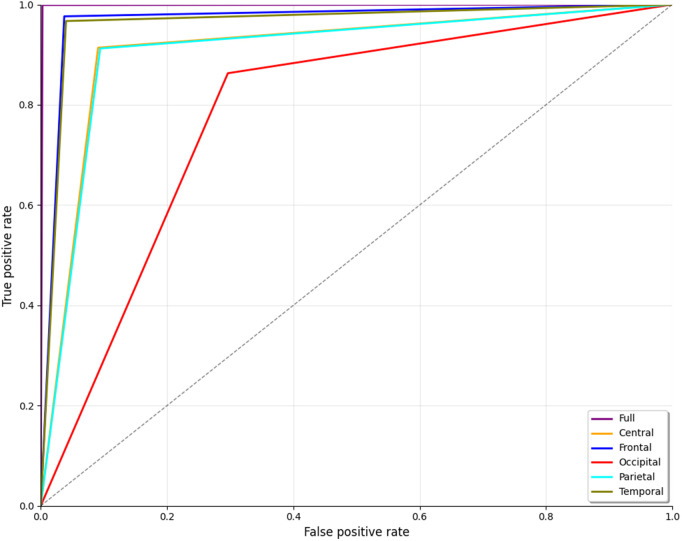
ROC curve for ScZ vs. HC classification for RepOD dataset.

The ROC curve is an illustration that plots the true positive rate on the y-axis and the false positive rate on the x-axis. The curve for the full brain region, represented by the purple line in Fig 9, demonstrates a nearly flawless classification performance. The strong elevation and position in the top-left corner of the graph signify a classifier that attains both high sensitivity (accurately identifying ScZ) and a minimal false positive rate (reducing erroneous identification). The frontal region, shown by the blue line, is close to the curve for the full brain lobe. This indicates that the frontal lobe data are highly informative for distinguishing. Other brain regions, including the parietal (cyan) and central (orange), showed moderate performance. Despite maintaining relatively high true positive rates, the performance variability indicates that these regions may not be as dependable for differentiating ScZ from HC as the entire brain or frontal region. Additionally, the temporal (olive) regions show better performance compared to the other regions, as they are closer to the frontal curve. Conversely, the occipital region (shown by the red line) demonstrates significantly inferior performance. This line diverges from the top-left corner, signifying a higher false positive rate. Overall, the ROC curve in Fig 9 indicates that the entire brain and frontal regions provide optimal performance. The temporal region demonstrates significant significance; although other regions show potential, their performance varies, with the occipital region notably under performing in accurate classifications.

**Fig 10:**
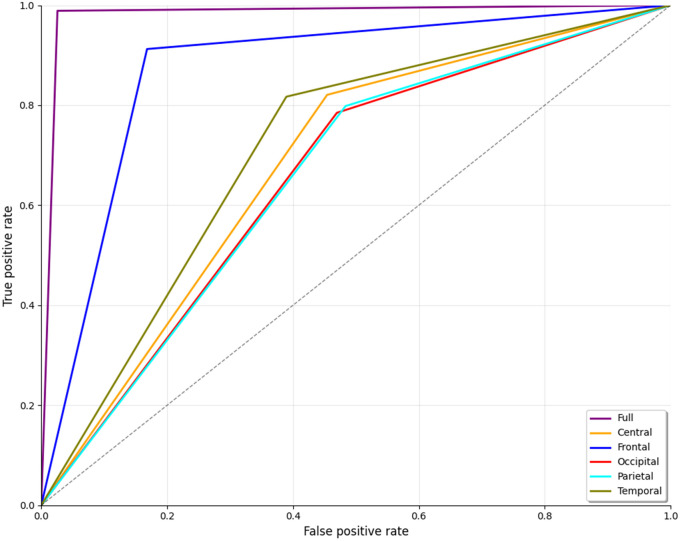
ROC curve for ScZ vs. HC classification for kaggle basic sensory task dataset.

The ROC curve shown in Fig 10 illustrates the classifier’s performance across different brain regions for distinguishing ScZ vs HC for the Kaggle dataset. Here again, the frontal region represented by the blue line also shows excellent performance, with a curve closely following the full brain analysis. Other brain regions, including the central region (orange) and parietal(cyan) regions, demonstrate balance performance. In contrast, the occipital region (shown by the red line) has poor outcomes in comparison. The deviation from the top-left corner of the graph indicates a high false positive rate and reduced sensitivity. A noticeable thing is that the temporal (olive) regions showed better performance compared to the other regions in the repOD dataset; this time, it shows a significantly lower performance, like the central and parietal lobes. This line position indicates that the temporal lobe is significant to overall outcomes, with areas providing vital information, although less accurately than the entire brain or frontal regions.

Overall, this study presents an approach that combines an STFT-based mel-spectrogram image and a CNN to identify critical brain lobes as biomarkers for distinguishing ScZ from HC using EEG data. The EEG channels were grouped according to their corresponding brain lobes to facilitate targeted analysis. Using STFT, the EEG signals were then turned into mel-spectrograms, which showed brain activity in terms of time and frequency mel bins. These mel-spectrogram images are put into a CNN model to classify them. The frontal lobe demonstrated the highest accuracy in both datasets; conversely, the occipital lobe has the lowest, consistent with the discovery of lobe-specific biomarkers for the detection of ScZ.

Each brain region uniquely contributes to the diagnostic process, and acknowledging these contributions enhances the accuracy and reliability of EEG-based ScZ detection [58,60–62]. The frontal lobe is critical for executive functions, which include higher-order cognitive processes such as decision-making, problem-solving, planning, and regulating behaviour. In patients with ScZ, EEG recordings from the frontal region often show altered brain activity, specifically an increase in beta power and a decrease in theta and delta power. The changes in these brainwave frequencies suggest a disruption in the mental processes required for effective functioning. Our study aligns with these findings, as we achieved the highest prediction accuracy when using EEG data from the frontal lobe across both datasets. These insights highlight the importance of targeting the frontal lobe in both diagnosis and therapeutic approaches for ScZ. Then, the temporal lobe, especially the hippocampus, is a bit critical for memory and cognitive skills, both of which are profoundly impacted in ScZ. EEG studies have indicated that ScZ patients often suffer lower alpha and beta power in the temporal areas, combined with increased theta and delta activity. We noticed that using EEG data from the temporal region offers the second-best classification accuracies for these two datasets, showing the one major significance of temporal lobe activity in distinguishing between ScZ and HC. The parietal lobe is essential for the integration of sensory information and for the facilitation of spatial navigation, taste, texture, and temperature. In patients with ScZ, EEG data from the parietal region frequently exhibit an increase in alpha activity and a decrease in theta activity. Our study attained a moderate classification accuracy across parietal regions investigated for ScZ classification, which is almost similar to central lobe accuracy. Besides, the central lobe Primary ScZ symptoms are less frequently linked to EEG signals from the sensorimotor cortex and other central brain areas. These areas may continue to show altered rhythms and other brain rhythm abnormalities, though, which might prove an indication of more widespread neural network changes in ScZs. This is demonstrated in our research as well, as we used this area of the brain to get the third-highest classification accuracy. Finally, the occipital lobe, which is mostly responsible for visual processing, shows less prominent changes in the early stages of ScZ. However, as the condition advances, EEG investigations have revealed that patients with ScZ may have a drop in alpha activity and an increase in slow-wave activity in the occipital areas. It suggests that the occipital lobe plays a less significant role in the development of ScZ. Our findings support this hypothesis, since we noticed that there were fewer improvements in classification accuracy when occipital EEG data was used, suggesting that less consideration is given to occipital activity for early and precise ScZ diagnosis.

### Ablation experiments

To make an informed decision, we have explored various investigations into different strategies that affect the performance of models.

In Table 5, it is clearly seen that the proposed STFT-based mel spectrogram achieves the highest accuracy of 99.82% with the CNN model. Subsequently, a traditional spectrogram-based CNN model yielded a closer result of 99.81% accuracy; however, this approach has already been explored by several researchers. On the other hand, we underwent more complex transformations, such as the Hilbert-Huang transform (HHT) and continuous wavelet scalograms, which resulted in a decrease in accuracy at 79.71% and 84.32%, respectively. We also explored the Vision Transformer (ViT) with a spectrogram that has an accuracy of 95.67% and the ViT + CNN combined with a spectrogram that has an accuracy of 96.95%. This indicates that image representation choice significantly influences model accuracy, and using deep learning with mel-spectrograms substantially enhances performance.

**Table 5:** Comparison of using different image generation techniques in different models in repOD dataset.

Image generation technique	Model	Accuracy (%)
STFT based spectrogram	CNN	99.81
STFT based spectrogram	ViT	95.67
STFT based spectrogram	ViT + CNN	96.95
Continuous wavelet transform based Scalogram	CNN	84.32
Continuous wavelet transform based Scalogram	ViT	87.23
Hilbert-Huang transform (HHT) based hilbert spectrogram	CNN	79.71
**Proposed STFT based mel spectrogram**	**CNN**	**99.82**

“ViT” indicates Vision Transformer.

### Comapring with Existing Method

To provide a thorough and insightful comparison between our proposed framework and the leading state-of-the-art (SoA) methodologies, we have used the RepOD and Kaggle sensory datasets as standards. In Tables 6 and 7, the side-by-side comparison has been carefully designed to not only showcase the strengths of our framework but also emphasize the specific advancements and improvements it offers over conventional methods. This analysis serves as a critical tool for validating the effectiveness of our method and positioning it as a meaningful refinement within the current landscape of research.

**Table 6:** Comparison with existing studies on ScZ vs. HC classification for RepOD dataset.

Study	Method	Segmentation	Validation	Accuracy
Buettner et al. [29]	RF	—	10-fold	96.77%
Vazquez et al. [28]	RF	644 segment per patient	7-fold	97.2%
De et al. [23]	SVM	—	—	89.00%
Das et al. [9]	SVM (Cubic)	—	—	98.9%
Jahmunah et al. [22]	SVM-RBF	6250 point samples	10-fold	92.91%
Siuly et al. [24]	Deep Feature based SVM	—	10-fold	99.23%
Shalbaf et al. [25]	ResNet-18-SVM	5	10-fold	98.60%
Aziz et al. [4]	K-nearest neighbors (KNN)	20s	10-fold	99.4%
Baygin et al. [26]	KNN	—	10-fold	99.47%
Haider et al. [30]	PSDL	225 segment per channel	—	89.12%
Gosala et al. [31]	PSDL	5s	5-fold	99.25%
Agarwal et al. [27]	Boosted trees classifier	4	—	99.24%
Li et al. [32]	Microstate semantic model	—	10-fold	97.2%
Sahu et al. [37]	RDCGRU	5 s	—	88.88%
Shoeibi et al. [63]	CNN-LSTM	—	10-fold	99.25%
Lillo et al. [18]	CNN	—	—	93%
Goker et al. [16]	1D CNN	5 min	—	98.76%
Guo et al. [45]	Three-dimensional Convolutional Neural Network (3DCNN)	4 s	10-fold	99.46%
Latreche et al. [34]	Ten-layer convolutional neural network(CNN)	—	10-fold	99.18%
**Proposed work**	**STFT based mel spectrogram CNN**	**3 s**	**10-fold**	**99.82%**

“—” indicates not reported in the paper.

**Table 7:** Comparison with existing studies on ScZ vs. HC classification for kaggle basic Sensory ask dataset.

Study	Method	Segmentation	Validation	Accuracy
Barros et al. [8]	CNN	—	10-fold	78 ± 8%
Guo et al. [21]	CNN	3 s	8:2 split	92%
Srinivasan et al. [64]	CNN+ LSTM	—	10 fold	98.2%
Ellis et al. [65]	1D CNN+LSTM	25 s	10-fold	75.9%
Sahu et al. [43]	Separable convolution attention network (SCZ-SCAN) via 2-D scalogram	4 s	—	95.00%
Siuly et al. [3]	EMD	—	10-fold	89.59%
Khare et al. [66]	RVMD and OELM	—	10-fold	92.93%
Khare et al. [38]	SPWVD-based TFR and CNN	3s	10-fold	93.36%
Ko et al. [17]	VGGNet- based CNN model	—	10-fold	93.2%
**Proposed work**	**STFT based mel spectrogram CNN**	**3 s**	**10-fold**	**98.31%**

“—” indicates not reported in the paper.

In Table 6, it is observed that, early studies often relied on conventional ML classifiers combined with handcrafted EEG features. For instance, Support Vector Machines (SVM) and k-nearest neighbors (KNN) proved unexpectedly competitive, achieving accuracies above 98% in several cases. Das et al. [9] demonstrated that an SVM model could achieve nearly 98.9%, while Baygin et al. [26] reported 99.47% with KNN. Siuly et al. [24] took a route, integrating deep feature extraction with an SVM classifier and achieving 99.23%. These findings highlight the value of leveraging the feature representation strengths of CNNs while preserving the stable generalization power of classic classifiers. Deep learning models—particularly CNNs and recurrent neural networks—have consistently achieved state-of-the-art results. Guo et al. [45] advanced this further with a 3D CNN architecture designed, achieving 99.46%, one of the highest performances across the literature. Similarly, Latreche et al. [34] employed a ten-layer CNN that reached 99.18%. Beyond raw signal processing, spectral and time-frequency methods remain valuable for uncovering discriminative patterns. Power Spectral Density-based Learning (PSDL), for instance, showed mixed outcomes: Haider et al. [30] reported a modest 89.12%, whereas Gosala et al. [31] refined the same approach to achieve 99.25%.

Now, move on to the Kaggle dataset’s existing approaches. In Table 7, we can see that one notable trend is the use of time-frequency representations to capture the non-stationary characteristics of EEG signals better. For example, Siuly et al. [3] employed Empirical Mode Decomposition (EMD), achieving an accuracy nearing 90%. Building on this, Khare et al. [38] applied Recursive Variational Mode Decomposition (RVMD) combined with Orthogonal Extreme Learning Machines (OELM), pushing the accuracy higher to approximately 93%. Their subsequent work incorporated further boosting performance to over 93%. Parallel efforts by Guo et al. [21] also confirmed the utility of CNN models, reporting accuracy of 92%. Ellis et al. [65], for example, explored a hybrid 1D CNN and LSTM network with an accuracy of around 76%. Ko et al. [17] VGGNet-based CNN demonstrated reliable performance with approximately 93% accuracy.

Our proposed work in this line of research has taken time-frequency representation further by using STFT-based Mel spectrograms as inputs to CNNs. This approach achieved an outstanding 99.82% on the RepOD dataset and 98.31% for the Kaggle dataset, surpassing earlier frameworks and underscoring the strength of integrating signal decomposition with deep architectures. This trajectory suggests a future where clinically applicable EEG-based diagnostic tools for ScZ may be both feasible and reliable, and can be built through our system.

### Explainability analysis

The term “XAI” (Explainable Artificial Intelligence) describes methods and techniques that make the decisions made by AI and machine learning models clear, understandable, and interpretable to humans. In recent years, XAI has evolved as a tremendous method for model explainability. XAI has become essential in understanding, validating, and trusting machine learning models, especially in sensitive domains like healthcare. In our study we have introduced three popular XAI techniques to explain the result outcomes. We performed a comprehensive explainability analysis on one representative fold across all subjects in both datasets, as our results remained very consistent across all folds, ensuring that the interpretability insights accurately capture the model’s decision-making patterns.

#### LIME.

LIME (Local Interpretable Model-agnostic Explanations) basically works by approximating the complex CNN’s behavior locally with an interpretable, simpler model, such as a linear regression, around a specific prediction instance. LIME is applied to individual mel-spectrogram images to generate heatmap-like explanations that reveal which frequency bands and time windows most influence the model’s predictions. This detailed analysis often shows that certain frequency rhythms, like altered gamma or theta bands, consistently stand out as important markers for identifying ScZ. Essentially, LIME breaks the mel-spectrogram into segments and visually indicates which parts of the image contribute positively or negatively to the model’s decision, making the prediction more understandable and transparent.

**Fig 11:**
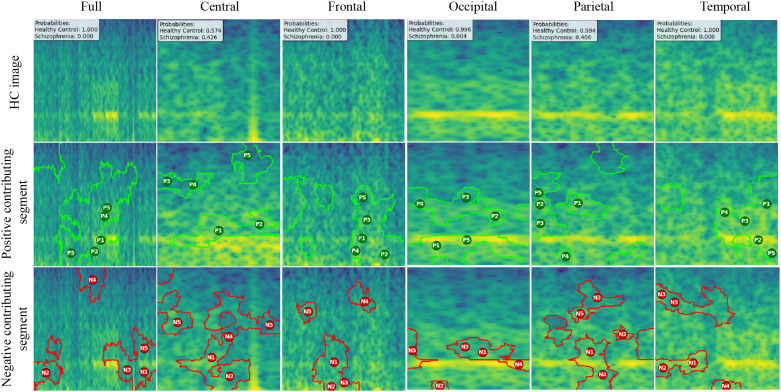
RepOD dataset LIME contributing segment in ScZ vs. HC classification for HC images in first segment for different brain regions.

**Fig 12:**
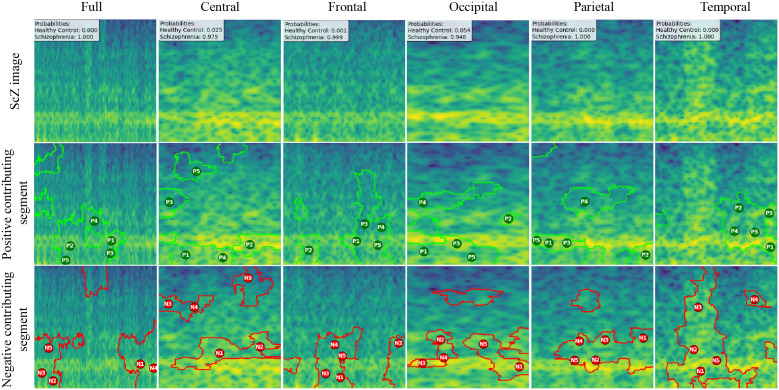
RepOD dataset LIME contributing segment in ScZ vs. HC classification for ScZ images in first segment for different brain regions.

**Fig 13:**
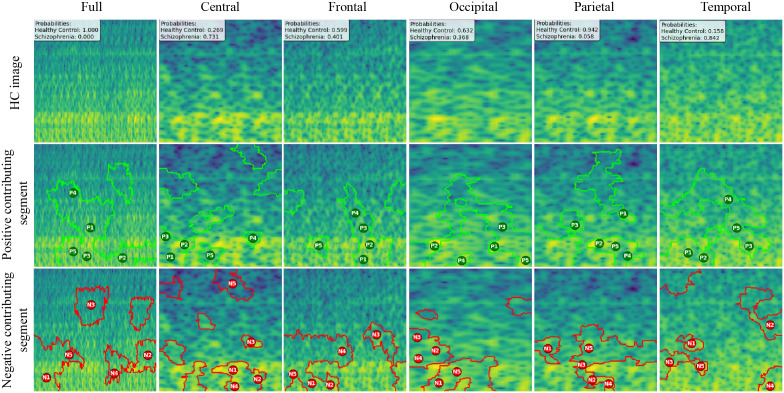
Kaggle dataset LIME contributing segment in ScZ vs. HC classification for HC images in first segment for different brain regions.

**Fig 14:**
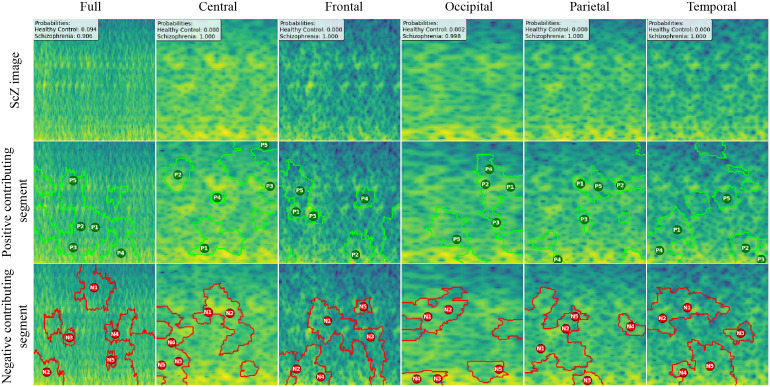
Kaggle dataset LIME contributing segment in ScZ vs. HC classification for ScZ images in first segment for different brain regions.

We created LIME visualizations for the full brain region as well as for five individual brain regions. For two different datasets, we have done the LIME visualizations. The LIME explanations for all five brain regions, along with the full region, are shown in Fig 11 and 12 for the repOD dataset. Fig 11 show healthy images for the first image after segmentation, and Fig 12 shows the corresponding first image of the ScZ patient image, which was classified by our CNN model across different brain regions. It is clearly observed which portion of the image is contributing positively and negatively to the disease classification. It basically identified a region as P1, P2, P3, etc, which means they are positively contributing to the model outcomes, and N1, N2, N3, etc, that negatively contribute to the model. Here, P1 and N1 are denoting the most contributing, then P2, N2, and so on. It is seen that the positive contributing and negative contributing segments in an image are different. When one segment of a ScZ image is positively predicted as ScZ, a different portion of the image is predicted as HC. Similarly, Figures 13 and 14 demonstrate the LIME explanations for all five regions, along with the full region for the Kaggle dataset. A similar pattern is consistently seen across all LIME explanations in both datasets.

These findings can be understood from various perspectives. It is seen that, in the full region for repOD, the positive contributing segment in the healthy image is ranked as $P_{1}>P_{2}>P_{3}$, etc, and in the Kaggle dataset is $P_{1}>P_{2}>P_{3}$ etc. The contributing segment portion is almost the same in both datasets. So, it is clearly observed which portion is reasonable for diagnosis of ScZ or not. This suggests that our model accurately predicts outcomes and identifies important features effectively. Since the input images are time-frequency mel spectrograms with time on the x-axis and frequency on the y-axis (filtered between 0.5 and 45 Hz), we notice that segments contributing to the ScZ classification are mainly located in the lower frequency bands in both datasets. These correspond to the delta and theta bands, which are dominant during deep, dreamless sleep and are also present in infants. Theta bands are associated with deep relaxation and meditation [60,61]. This insight may help clinicians in diagnosing and treating ScZ. On the other hand, the HC images were mainly associated with alpha and beta bands [60,62], as seen in the image prediction, contributing positively to a higher portion of the image. These observations provide helpful information for doctors in their diagnostic decision-making process.

From the earlier results, it was evident that the frontal brain region provided the highest accuracy in classifying ScZ, while the occipital lobe showed the lowest accuracy [58,60,62].

A clear pattern emerges from these images: the segments that contribute positively and negatively to the model’s predictions in the frontal region are very similar and closely align with those observed in the full brain region’s LIME explanations. This close alignment suggests that the frontal region carries much of the predictive information that drives the model’s success, supporting its importance in ScZ diagnosis. The high accuracy achieved with the frontal region demonstrates that it captures critical features related to the disorder. In contrast, the key contributing segments of the occipital region’s mel-spectrogram are spatially far away from those identified in the full brain region. This finding suggests that the occipital lobe has a weaker connection to the features important for diagnosing ScZ according to our model. The frontal lobe plays a critical role in capturing neural patterns linked to ScZ, making it an influential region for model predictions [60]. On the other hand, the occipital lobe’s contribution is minimal or even contrasting, indicating it is less relevant to the diagnosis [62]. From a clinical perspective, practitioners can focus more on frontal lobe-specific treatment procedures when interpreting EEG data for ScZ diagnosis, potentially improving the accuracy and reliability of their assessments. At the same time, recognizing that the occipital region contributes less can help streamline diagnostic processes and avoid unnecessary emphasis on less informative brain areas. Ultimately, these insights can guide targeted treatment strategies and support better decision-making in managing ScZ.

#### SHAP.

SHAP (SHapley Additive exPlanations) is a technique used to explain the predictions of machine learning models by assigning a contribution value to each feature. SHAP basically ranked the feature importance of the model which is why its transparency and fairness have made SHAP a prevalent tool for decision-making in fields like healthcare.

**Fig 15:**
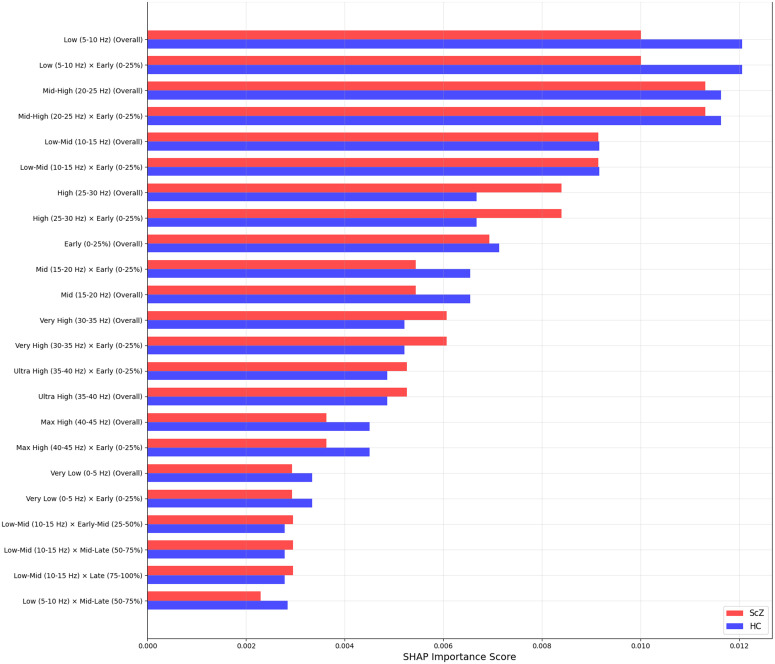
SHAP importance rankings for repOD dataset for the full region.

**Fig 16:**
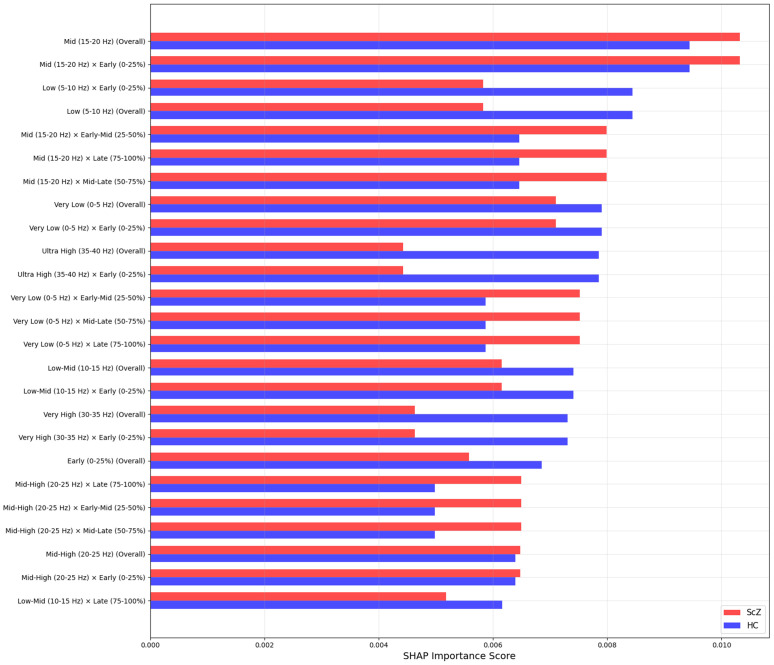
SHAP importance rankings for Kaggle basic sensory task dataset for the full region.

We applied SHAP only for the full region to identify the most important features in the model’s decisions. As we filtered the image from 0.5 to 45 Hz. Fig 15 and 16 shows important features from the full brain region, in classification tasks, ScZ vs HC. It produces feature importance by ranking time segments and frequency range. It is observed that the full brain SHAP ranking has similar feature importance in both datasets.

It is clearly visible in the Fig 15 that in the repOD dataset, the full region with the most influential frequency band spans the mid-level (20–25 Hz) and low-level (0–5 Hz) frequency ranges for ScZ diagnosis. Conversely, for HCs, the range is also the same. LIME visualizations also confirm this range. Then, a slightly varied range is identified in the basic Kaggle dataset in Fig 16. It is visible that, for ScZ, the range is between 15–20 Hz and 0–5 Hz. Furthermore, for HCs, the range lies mostly between 15–20 Hz and 5–10 Hz. These are nearly similar in both datasets and also establish the outcomes of other XAI techniques. We did not explicitly account for batch effects in our data processing or analysis. The slight differences observed in SHAP feature ranking between the RepOD and Kaggle datasets are likely attributable to inherent differences in data acquisition protocols, recording conditions, experimental paradigms, and participant populations.

### Grad-CAM

Grad-CAM (Gradient-weighted Class Activation Mapping) is a widespread approach utilized to visualize and analyze the decision-making process of CNNs by spotlighting which regions of an input image were most influential for a particular prediction.

**Fig 17:**
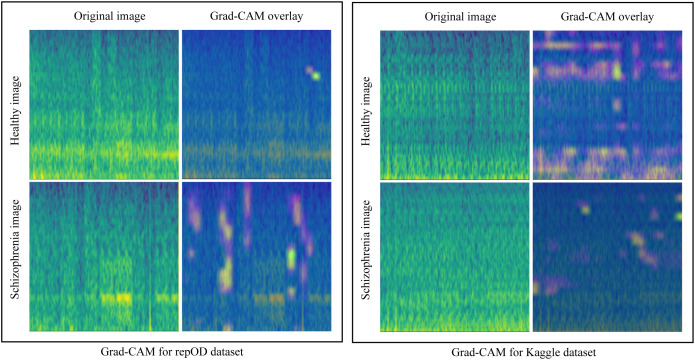
Grad-CAM for the repOD dataset and Kaggle basic sensory task dataset for the full region.

To identify the most influential mel-spectrogram features in the model’s decisions, we applied the Grad-CAM method only for the full region. Fig 17 presents a sample HC and ScZ mel-spectrogram images from the full brain lobe, along with its corresponding Grad-CAM visualizations. It is observed that the full brain Grad-CAM image overlay has significant regions with a high heatmap area on both occasions [60].

## Conclusion

In this research, we have developed a framework to identify critical brain lobes for the detection of ScZ from HC subjects using EEG data. Our approach begins by segmenting the EEG signals, followed by transforming these segments into mel-spectrogram images using STFT. These mel-spectrogram images are then classified using a CNN model. We evaluated our model on two datasets to bring clarity and acceptance of our proposed model. We applied this framework to the full brain lobe as well as five distinct brain lobes, aiming to pinpoint the regions most significant for ScZ detection. Through analyzing EEG data from publicly available two datasets, we have demonstrated a high accuracy in both datasets, where a mel-spectrogram image is used for the first time in this domain of research. We have also discovered that the frontal lobe showed the most significant changes across both the dataset, followed by the temporal lobes. The occipital lobe shows less importance in the diagnosis accuracy. These findings emphasize the importance of targeting specific brain lobes when developing diagnostic and monitoring tools for ScZ. To enhance the interpretability of our results and provide meaningful insights, LIME, SHAP, and Grad-CAM techniques is applied to improve the transparency of our framework further. By incorporating XAI techniques, our research not only advances the accuracy of ScZ detection but also provides actionable insights for clinicians and researchers to interpret the model’s decisions, ultimately contributing to more informed and effective healthcare solutions.

Research to date has largely focused on applying a single methodology across multiple diseases. Further studies should investigate the applicability of EEG in identifying and monitoring additional neurodegenerative disorders, including Parkinson’s Disease, Huntington’s Disease, and epilepsy, to evaluate the generalizability of our methodology. Therefore, there is an opportunity to extend and validate this approach by implementing it in the context of other disorders. Integrating multimodal data, including structural MRI and genetic information, could boost the model’s predictive capability and provide a more thorough explanation of disease causes. Although we have already evaluated our technique on two distinct datasets, testing it on additional large EEG datasets for ScZ will enhance its generalization. Testing across diverse populations and recording conditions will be essential to establish the robustness and reliability of our approach for real-world clinical deployment. As we did not explicitly account for batch effects in our data processing or analysis. We consider implementing batch correction techniques and conducting systematic investigation of dataset-specific effects as future work. Additionally, collaboration with clinical institutions to compile larger prospective datasets will be a priority to advance the translation of this technology into practical diagnostic tools. Ultimately, the incorporation of EEG-based frameworks into clinical practice represents a potential breakthrough. Executing our methodology in practical environments and performing longitudinal research could result in critical insights, facilitating the enhancement of the technology and ultimately advancing personalized, effective healthcare solutions for neurological diseases.
